# Potentiometric Performance of Ion-Selective Electrodes Based on Polyaniline and Chelating Agents: Detection of Fe^2+^ or Fe^3+^ Ions

**DOI:** 10.3390/bios12070446

**Published:** 2022-06-23

**Authors:** Rimeh Ismail, Ivana Šeděnková, Zulfiya Černochová, Iryna Romanenko, Ognen Pop-Georgievski, Martin Hrubý, Elena Tomšík

**Affiliations:** Institute of Macromolecular Chemistry AS CR, Heyrovského Nám. 2, 162 06 Prague, Czech Republic; ismail@imc.cas.cz (R.I.); sedenkova@imc.cas.cz (I.Š.); cernochova@imc.cas.cz (Z.Č.); romanenko@imc.cas.cz (I.R.); georgievski@imc.cas.cz (O.P.-G.); mhruby@centrum.cz (M.H.)

**Keywords:** sensor of Fe^2+^ or Fe^3+^ ions, non-biofouling layer, poly(2-methyl-2-oxazoline)s, potentiometry, analysis

## Abstract

We constructed a sensor for the determination of Fe^2+^ and/or Fe^3+^ ions that consists of a polyaniline layer as an ion-to-electron transducer; on top of it, chelating molecules are deposited (which can selectively chelate specific ions) and protected with a non-biofouling poly(2-methyl-2-oxazoline)s layer. We have shown that our potentiometric sensing layers show a rapid response to the presence of Fe^2+^ or Fe^3+^ ions, do not experience interference with other ions (such as Cu^2+^), and work in a biological environment in the presence of bovine serum albumin (as a model serum protein). The sensing layers detect iron ions in the concentration range from 5 nM to 50 µM.

## 1. Introduction

Over the last several years, sensors and biosensors have received much attention and have been developed with the growth of biosciences, chemistry, and engineering [1,2]. They still remain an active field of research and development. Moreover, they have been widely used in different areas, including biomedical, diagnostic treatment, and biosensing applications [3,4]. In addition, conducting polymers have received considerable attention. They can be employed as excellent materials for chemical or electrochemical sensors as ion-to-electron transducers [5,6,7]. Polyaniline (PANI) remains one of the most promising conducting polymers due to its low cost, excellent pseudocapacitive performance, ease of processing, and remarkable environmental stability [8,9,10]. PANI has been extensively studied as a good candidate in electrode materials [11,12,13]. In this context, research on the use of PANI in the fabrication of efficient sensors and biosensors has been published [14,15,16,17,18,19,20]. Alcohol sensors have been studied by Rishi Pal et al. [16]. PANI/graphene composites were prepared via a simple chemical oxidation method with four different concentrations of graphene (2, 4, 6, and 8 wt%) in PANI. The results showed that the PANI/graphene composite prepared with 8 wt% of graphene indicated the highest sensing response (~61.50% at 100 ppm) and the lowest response time (60 s). Delloula et al. [11] prepared NiFe-PANI by an electrochemical technique for glucose detection and demonstrated that the fabricated sensor exhibited excellent selectivity, reproductivity, and stability for glucose detection compared to a Ni(NPs)-PANI electrode. Creatine detection was investigated by Sriramprabha et al. [17] using an Fe_2_O_3_/PANI supramolecular nanocomposite. Recently, Tomsik et al. [18] synthesized a superhydrophobic conductive polyaniline film by the electrochemical method. Their results demonstrated that the proposed nanocomposite can be used as a solid contact for ion-selective electrodes and as a sensor for the detection of various ions in critical care medicine. Moreover, the authors proved that the film showed high stability in the long-term cycle (5000 cycles). The measurements also revealed the selective detection of H^+^ by the perfluorinated polyaniline film, even in the presence of interfering ions and/or human serum albumin. Chen et al. [19] developed electrodes (ultrathin polyaniline nanosheets) for sensitive and selective dopamine detection. The authors reported that the fabricated nanocomposite exhibited excellent selectivity, reproducibility, and stability.

Among the diverse elements in biological systems, iron is recognized as one of the most essential elements in the human body. However, both deficiency and excess iron can cause serious diseases. Iron deficiency might cause anemia [20]. Excess iron in the body can lead to disorders (hemochromatosis) [21]. It is necessary to maintain the normal level of iron concentration in the human body to avoid potential health hazards. Iron in the diet is present mainly as Fe^3+^. In the small intestine, it is reduced by the intestinal mucosa to Fe^2+^ and absorbed. In blood plasma, iron is transported as an Fe^3+^-transferrin complex. Intracellularly both Fe^2+^ and Fe^3+^ in proteins are present with an oxidation state dependent on the particular case. In milk, saliva, tears, and nasal secretions, Fe^3+^-lactoferrin is the most abundant iron form. Concerning this article, extracellularly, only Fe^3+^ and not Fe^2+^ is a relevant oxidation state in body fluids. As a result, the detection of iron in the biological system is crucial. Several techniques, such as atomic absorption spectroscopy (AAS) [21], inductively coupled plasma (ICP) [22], etc., have been used for detecting iron ions. Nevertheless, their expensive instrumentation and complicated and time-consuming procedures require highly skilled operators and restrict their investigation. Thus, potentiometric ion-selective electrodes (ISE) have been recognized as an attractive approach due to their simplicity, selectivity, and low cost. It was also reported that ISE was used for the determination of cations/anions and was also used for pharmaceutical compounds [23,24,25,26]. Currently, there have been only limited reports on the potentiometric detection of Fe^3+^ ions in the literature [27,28,29]. Abdallah et al. developed a new potentiometric sensor for the quantification of Fe^2+^ ions via an MWCNTs-Gemifloxacin composite in the range of 10^−2^ mol L^−1^ to 10^−8^ mol L^−1^ with a Nernstian slope of 30.37 ± 0.3 (mV/decade). The author reported that the fabricated electrode can be used in multivitamins tablets, tap water, and milk samples with good recovery (94.00–102.00%). Mizani et al. [28] proposed a potentiometric sensor based on 9-ethylacenaphtho [1.2-b] quinoxaline (EANQ) for the determination of iron ions in pharmaceutical samples and water samples. The proposed sensor displays a fast response time of 25s, with a long lifetime of 5 months, selectivity (on the order of 10^−4^ and 10^−5^) over a number of cations, and it exhibited a Nernstian slope of 19.5 ± 0.3 mV/decade of activity over Fe^3+^ ion concentration ranges of 2.3 × 10^−7^–5 × 10^−2^ M with very low limits of detection (9.6 × 10^−8^ M).

In the current work, ion-selective sensors based on PANI and chelating agents were designed to detect iron(III) and/or iron(II), and a potentiometric technique was successfully applied in order to sense these ions in the relevant biological medium. First, the PANI layer was deposited on graphite carbon by chemical oxidative polymerization, then chelating molecules (2,2 bipyridyl, 1,10-phenanthroline-5-amine, or 8-hydroxyquinoline) were deposited on top of PANI@graphite carbon by drop-casting. Cyclic voltammetry in a non-aqueous solution was applied in order to fix the chelating layer. Finally, a non-biofouling layer was deposited on top of the sensing layer in order to protect it from a nonspecific interaction that could be caused by proteins, even directly in the complex biological environment. Poly(2-methyl-2-oxazoline)s was chosen as the non-biofouling layer material. It has been extensively used in biomedical applications [30] such as in drug delivery systems, bio-fabrication, and the design of synthetic bio-interfaces [31] because of its biocompatibility and stealth properties [32].

## 2. Materials and Methods

Sodium chloride (Lach-ner, Neratovice, Czech Republic), ammonium peroxodisulfate (Lach-ner), formic acid (Sigma Aldrich, Prague, Czech Republic), analytic grade aniline (Lach-ner), and poly(2-methyl-2-oxazoline)s were synthesized according to Ref. [33]. The 2,2 bipyridyl, 1,10-phenanthroline-5-amine and 8-hydroxyquinoline (Sigma Aldrich, Czech Republic) used in the experiments were used as received without further purification.

Iron (III)-chloride hexahydrate (Sigma Aldrich, Czech Republic), iron (II) chloride (Sigma Aldrich, Czech Republic), and copper (II) chloride (Sigma Aldrich, Czech Republic) were used to study the sensing ability of the electrodes.

### 2.1. Synthesis of Polyaniline/Graphite Carbon Rod (PANI@G.C)

**Scheme 1:**
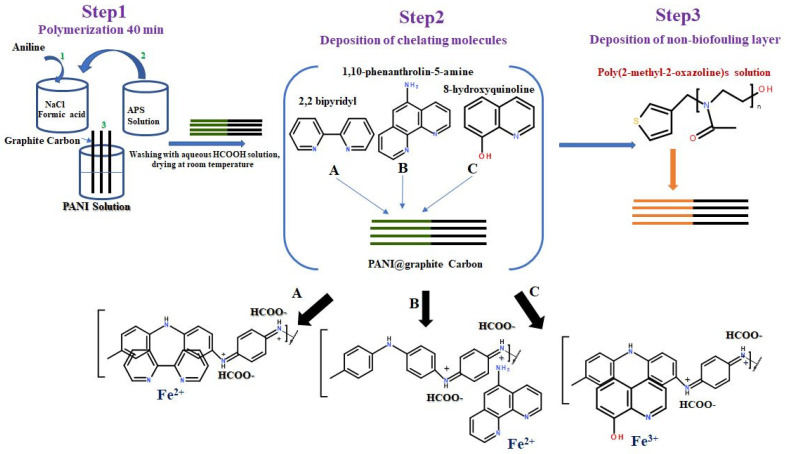
Preparation of sensing layer: chemical deposition of PANI layer, then drop-casting deposition of the chelating molecule, and finally deposition of non-biofouling poly(2-methyl-2-oxazoline)s layer.

The synthesis was performed under conditions as follows: First, 6.09 g of NaCl was dissolved in 20.3 mL of distilled water, then concentrated formic acid (HCOOH, 4.7 mL) was added to the solution. The prepared solution was purged with nitrogen for 5 min. After that, aniline (0.1 mL, 4.0 × 10^−2^ M) was dissolved in the mixture, and graphite carbon rods (G.C) were placed in the solution (see Scheme 1). The aqueous solution of ammonium peroxydisulfate (APS) (0.228 g, 4.0 × 10^−2^ M) was finally added to the mixture as an oxidant. The ratio of the monomer to oxidant was 1:1. The polymerization was performed for 40 min at room temperature. The obtained electrodes were washed carefully with 5.0 M aqueous formic acid solution (HCOOH) and dried at room temperature.

### 2.2. Deposition of Chelating Molecules on PANI@G.C Electrodes

The state-of-the-art of iron detection is the application of chelating low-molecular-weight molecules as a layer on top of the PANI layer. Iron is a transition metal of the fourth period. Iron ions occur in biological systems, mainly in the oxidation states 2^+^ and 3^+^. According to Pearson’s hard and soft (Lewis) acids and bases (HSAB) concept, iron (III) is a “hard” Lewis acid that could be coordinated by oxygen-containing ligands (hydroxide, phenolate, and carboxylate), and iron (II) is a “soft” Lewis acid and tends to coordinate nitrogen ligands (such as amines and imidazole) [34]. Based on this knowledge, three chelating molecules were chosen: 2,2-bipyridyl and 1,10-phenanthroline-5-amine for chelating Fe^2+^ ions and 8-hydroxyquinoline for chelating Fe^3+^ ions.

As can be seen in Scheme 1, three different chelating molecules were applied on different PANI@G.C electrodes. Each chelating molecule (0.1 M) was dissolved in 1 mL of ethanol (Purity (p) > 99.8%) and then deposited on top of the PANI@G.C by drop-casting. The corresponding sensing electrodes were named accordingly (see Table 1):

The chelating layer was fixed by applying a cyclic voltammetry technique in the potential window from −0.1 V to 0.8 V vs. a Ag/AgCl reference electrode (scan rate 50 mV/s, 10 cycles) in acetonitrile.

### 2.3. Deposition of POX Layer on PANI@G.C Electrodes

A poly(2-methyl-2-oxazoline)s (POX) solution (1%) with a molecular weight of 2 kDa was placed on A-PANI@G.C, B-PANI@G.C, and C-PANI@G.C. The POX/(A, B, and C)-PANI@G.C electrodes were finally dried under ambient conditions (see Table 1). To fix the POX layer, the electrochemical cyclic voltammetry method was used in acetonitrile because POX does not dissolve in organic electrolytes.

### 2.4. Characterization Methods

To analyze the deposited polymer films and to prove the presence of iron in the sensing layer, different methods were applied: Raman spectroscopy, scanning electron microscopy (SEM), and electrochemical techniques (cyclic voltammetry and electrochemical impedance spectroscopy (EIS)).

The chemical structure was investigated by Raman spectroscopy, and two lasers were used (514 nm and 633 nm). Raman spectra were recorded with a Renishaw inVia microspectrometer equipped with a Leica DM LM microscope (Leica, Wetzlar, Germany). The 50× objective was used to focus the laser beam. The spectra were measured with two excitation lines, a 514 nm Ag laser and a 633 nm He-Ne laser with gratings of 2400 lines mm^–1^ and 1800 lines mm^–1^, respectively.

Spectroscopic ellipsometry (SE) was performed on a J.A. Woollam M-2000X ellipsometer (Lincoln, Nebraska, USA) operating in a rotating compensator mode at an angle of incidence (AOI) range of 58–71° (with a step of 1°) and a spectral range of *λ* = 250–1000 nm. One of the main problems associated with the SE analysis of thin absorbing coatings is the strong correlation between the thickness and the optical properties. To increase the precision of the PANI thickness determination utilizing spectroscopic ellipsometry, the optical dispersion function of the PANI polymer layer was determined by the interference enhancement method [35,36] on previously characterized SiO_2_ (1010.0 ± 1.5 nm)/Si substrates (samples of size 1 × 2 cm^2^ were cut from double-side polished silicon wafers (CZ, orientation <100>, B-doped, resistivity 15-25 Ω cm) with ~1 μm wet silicon dioxide (Siegert Wafer GmbH, Aachen, Germany)). In this case, a two-layer optical model was used consisting of the silicon substrate, a SiO_2_ and PANI layer, and ambient air. The presence of a thick transparent SiO_2_ layer between the silicon substrate and the polymer films causes damped interference oscillations in measured ellipsometric spectra (Ψ and Δ), enhancing the sensitivity of the measurement/data extraction to both film thickness and the film optical constants. The optical dispersion function of the PANI films was modeled with three Lorentz oscillators and one free carrier term, assuming an isotropic sample structure [37].

Dynamic light scattering (DLS) measurements were performed using the Zetasizer NanoZS instrument, model ZEN3600 (Malvern Instruments, Malvern, Worcestershire, UK). The size distribution of the polymeric assemblies in the polymer solutions (1.05 mg·mL^−1^ of POX 1.2 kDa, 1.75 mg·mL^−1^ of POX 2 kDa, and 4.1 mg·mL^−1^ of POX 4.7 kDa in deionized water) was carried at temperatures of 10, 15, 20, 25, 27, 29, 30, 31, 33, 35, 37, and 39 °C. The hydrodynamic radius (RH) was measured at a scattering angle of θ = 173°, and the data were processed with the Repes algorithm. All POX solutions were filtered before measurement using a 0.45 μm PVDF syringe filter.

The electron micrographs were obtained using a JEOL 6400 microscope (Tokyo, Japan).

The electrochemical characterization of the electrodes was performed in a two-electrode cell configuration using an AUTOLAB PGSTAT302N potentiostat with a FRA32M module and Nova 2.1 software. A Pt sheet (1.2 cm^2^) was used as the counter electrode, and Ag/AgCl (3 M KCl) was used as the reference electrode. The electrochemical measurements were carried out at room temperature in HCOOH (5 M), HCl (0.1 M), or in an aqueous solution of NaCl (0.1 M). For the antifouling test, bovine serum albumin (BSA) was added to the electrolyte during EIS measurements. Cyclic voltammetry was measured in the potential window from −0.1 to 0.8V vs. a Ag/AgCl reference electrode at 50 mV/s scans. Electrochemical impedance spectroscopy (EIS) was performed in the frequency range from 10 kHz to 0.1 Hz at open circuit potential (OCP).

The potential stability of electrodes was measured using a 6-channel high-input-impedance voltmeter with the input impedance of 10^10^ Ω (Lawson Laboratories, Malvern, PA, USA).

The potentiometric measurements were performed in 0.1 M NaCl or in the solution of PBS. The sensing electrode was immersed in the solution and waited until the potential drift was not detected. After that, iron ion was added (starting from the smallest concentration), the solution was stirred for 3 min, and the potential response was recorded after 4 min.

## 3. Results and Discussion

PANI is an appropriate semiconducting polymer to be applied as an ion-to-electron transducer. Based on the literature data, it is suggested to polymerize PANI under such conditions when the crystalline film is obtained [38]. Organized PANI chains provide good charge transfer in the bulk of the film and at the interface, as was proven previously by our group. The deposition of PANI film was performed in the presence of formic acid and Hofmeister ions that could weaken the water structure, which leads to the hydration of polymer chains. The reaction took place for 40 min in order to obtain a thin film and avoid the deposition of globular PANI formed in the bulk of the polymerization solution. The time of the deposition was chosen based on the ellipsometry measurement results. It was found that a PANI layer with a thickness of 80–100 nm was obtained during the 40 min of polymerization.

### 3.1. Raman Spectroscopy

**Figure 1:**
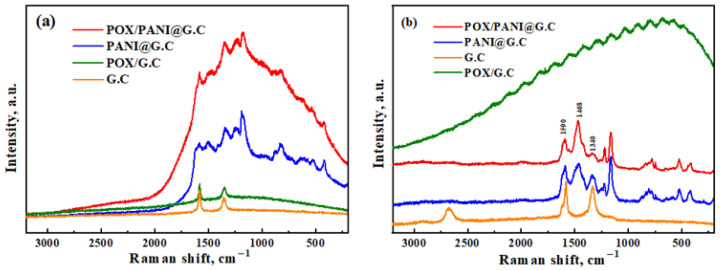
Raman spectra. The 514 nm (**a**) and 633 nm (**b**) excitation lines of G.C, POX @G.C, PANI @G.C, and POX/PANI@G.C.

Raman spectroscopy was applied to prove the structure of PANI and the deposition of a non-biofouling layer on top of the PANI. First, the individual spectra of two polymers were recorded, as presented in Figure 1a,b.

Figure 1a,b show the Raman spectra of samples obtained with the 514 nm and 633 nm excitation lines, respectively. As can be observed, the Raman spectra of the POX layer on the carbon electrode measured with the 514 nm excitation line did not reflect any bands connected with the vibrational states of POX. However, the fluorescence background of the spectrum increased due to the presence of POX. The fluorescence was then apparent in the spectra of POX covering the polyaniline layer prepared on the carbon electrode. This is indirect proof of the presence of POX. The fluorescence was more pronounced as the molecular weight of POX increased. For our purpose, the 2 kDa POX was chosen as the most suitable.

The Raman spectra recorded at the 633 nm excitation line are presented in Figure 1b. As the fluorescence of POX was suppressed, the red excitation laser could be used to study the molecular structure of polyaniline. It can be noticed that the intensity of the band at 1340 cm^–1^, connected with the stretching vibration of the polaronic C~N^+^ structure, in the spectrum of PANI on the carbon electrodes decreased after coating with POX. The intensity of the band located at about 1468 cm^–1^, linked to the C=N stretching in the imine sites of the emeraldine base, was higher. The development reflected the decrease in the polaron concentration in the polyaniline structure and the conversion to the base form due to the POX coating. This result demonstrates that the molecular weight of POX does not have any significance in the process.

It must be emphasized that the chemical structure of the chelating molecules is similar to PANI, which is why it is tough to identify the presence of these chelating agents in the composite structure of the sensing films. However, the application of Raman spectroscopy and electrochemical impedance spectroscopy was used to prove the presence of chelating molecules on top of PANI. The data for the Raman measurements are presented in SI Figures S3–S5. The Raman spectrum of the sample A-PANI@G.C (Figure S3) reflects the presence of 2,2-bipyridyl. The 2,2-bipyridyl molecule is in the resonance with the green excitation line 514 nm and, as such, the Raman bands are enhanced in the spectrum of the A-PANI@G.C and are presented at 1605 cm^–1^, 1490 cm^–1^, 1024 cm^–1^, and 662 cm^–1^.

The strong fluorescence prevailed in the Raman spectrum of excited 1,10-phenanthroline-5-amine (Figure S4). The increase in fluorescence was then mirrored in the spectrum of the sample of B-PANI @G.C.

The 8-hydroxylquinoline and the C-PANI@G.C were measured with the 514 nm excitation line as well as with the 633 nm and 785 nm excitation lines. In all three Raman spectra, the Raman bands of hydroxyquinoline were overlapped by the spectral features of PANI, and the presence of the 8-hydroxylquinoline could not be clearly confirmed (Figure S5).

### 3.2. Electrochemical Characterization

Electrochemical methods (cyclic voltammetry and EIS) were applied to prove the ability of PANI films to be used as ion-to-electron transducers. Moreover, the deposition of the POX layer did not deteriorate the electrochemical performance of PANI film, as presented below. Furthermore, the chelating molecules were able to chelate iron ions on one hand and, on the other hand, protect the PANI layer from direct interaction with the studied medium, which leads to the PANI layer only working as the ion-to-electron transducer.

#### 3.2.1. Cyclic Voltammetry

The cyclic voltammograms of polyaniline, prepared by chemical oxidative polymerization, were examined in the range from −0.1 to 0.8 V versus a Ag/AgCl reference electrode recorded at a 50 mV/s scan rate in the aqueous solution of HCl, HCOOH, and NaCl.

It is well-established that PANI can be electro-oxidized during a voltammetry potential sweep and exist in several forms that differ by their oxidative levels [39].

**Figure 2:**
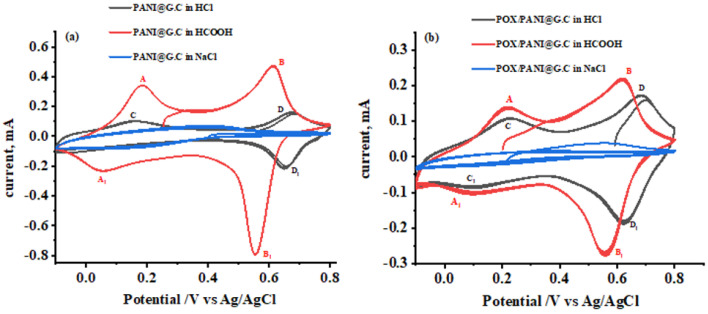
Cyclic voltammograms of PANI@G.C (**a**) and POX/PANI@G.C (**b**) were recorded at a 50 mV/s sweep rate in the aqueous solution of HCl, HCOOH, and NaCl.

It can be seen in Figure 2 that two redox pairs were observed (Figure 2a,b) on the CV voltammograms measured in HCOOH and HCl. Generally, the cyclic voltammograms of PANI film measured in an aqueous solution of acid consist of two main oxidation peaks. The first maximum, at about 0.2 V versus Ag/AgCl corresponds to the oxidation of leucoemeraldine to emeraldine, and the second maximum at the higher potential of 0.7 V is attributed to the oxidation of emeraldine to pernigraniline [39]. Thus, the first redox peaks (Figure 2a: A, A_1_—corresponding to E_1/2_ = 0.18–0.04 = 0.07 V, C—0.15 V vs. Ag/AgCl reference electrode, Figure 2b: A, A_1_—corresponding to E_1/2_ = 0.22–0.08 = 0.07 V, C, C_1_—E_1/2_ = 0.23–0.1 = 0.06 V vs. Ag/AgCl reference electrode) are commonly attributed to the oxidation of 50% of the monomer units of PANI film (oxidation of leucoemeraldine to emeraldine). The second redox peaks (Figure 2a: B, B_1_—corresponding to E_1/2_ = 0.61–0.55 = 0.03 V, D, D1—E_1/2_ = 0.67–0.65 = 0.01 V, Figure 2b: B, B1—corresponding to E_1/2_ = 0.61–0.55 = 0.03 V, D, D_1_—E_1/2_ = 0.68–0.62 = 0.03 V vs. Ag/AgCl reference electrode) correspond to the other half of the monomer units (emeraldine to pernigraniline). Markedly, no peaks associated with the redox processes of PANI were detected when the cyclic voltammetry measurements were performed in NaCl (neutral electrolyte), indicating that the redox processes of PANI are induced by the presence of acid (formic acid or hydrochloric acid) acting as a supporting electrolyte, and PANI in HCOOH more easily underwent redox processes than in HCl.

The deposition of the chelating agent and non-biofouling layer did not change the electrochemical activity of the PANI films, as can be seen in Figure 2b. The decrease in the current is explained by the presence of the non-biofouling layer on top of PANI, and part of PANI film does not participate in oxidation/reduction processes. These measurements provide evidence that the PANI layer could be used as an ion-to-electron transducer layer in neutral conditions and acidic ones. To further investigate the resistance of the individual layer and/or composite sensing layers, the electrochemical impedance spectroscopies were measured.

#### 3.2.2. Electrochemical Impedance Spectroscopy

**Figure 3:**
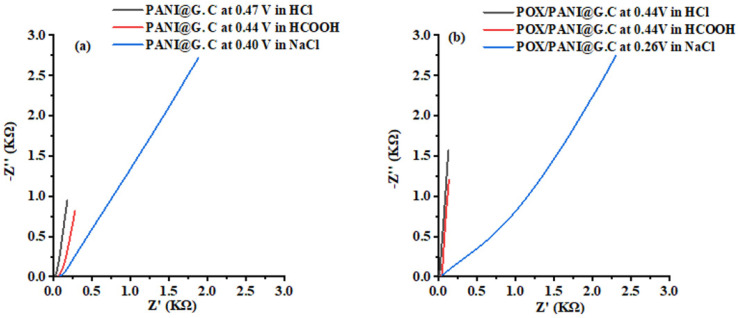
EIS of PANI@G.C (**a**) and POX/PANI@G.C (**b**) was measured at OCP in different electrolytes.

EIS is a unique technique to characterize the semiconducting film and prove its ability to conduct the electrons under different conditions. The results of the measurements are presented in Figure 3 for PANI@G.C and POX/PANI@G.C. The results of the EIS measurements for A-PANI@G.C, B-PANI@G.C, and C-PANI@G.C are presented in SI; see Figure S1.

The deposited PANI film had a lower resistance in the acidic medium compared to the neutral one, as recorded in Figure 3a. This finding correlates with the literature data [18]. The deposition of the chelating layers (see Figure S1) led to an increase in the resistance with the simultaneous appearance of the semicircle, which was connected with the poor conductivity of the chelating agents. These data show that we successfully attached chelating molecules to the PANI layer. Moreover, the deposition of a non-biofouling layer (POX) slightly increased the resistance of the final composite sensing layer films, confirming the presence of the non-biofouling layer. It must be emphasized that the EIS performance of sensing films in a neutral medium (with the presence of the non-biofouling layer) has a diffusional controlled nature (see Figure 3b), which means that the resistance is proportional to the diffusion of the ions through the protecting layer.

### 3.3. Dynamic Light Scattering Measurement (DLS)

The DLS technique was applied to study the solution of POXs (1.2 kDa, 2 kDa, and 4 kDa) in order to find the appropriate POX molecular weight and conditions when the polymer chains could be deposited on top of the sensing layer to form a uniform non-biofouling layer.

**Figure 4:**
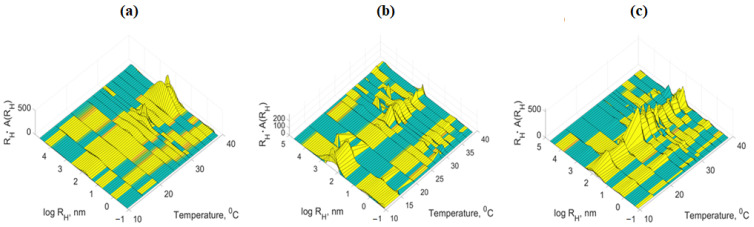
DLS results of POX 1200 (**a**), POX 2000 (**b**), and POX 4700 (**c**) dissolved in water at room temperature.

The self-assembly of POXs with various molecular weights (1.2 kDa, 2 kDa, and 4.7 kDa) was investigated by dynamic light scattering (DLS) (Figure 4a–c). The DLS results showed that below 25 °C the POXs formed particles with different size distributions (molecularly dissolved as soft globules), and when the temperature was raised above 25 °C, micelle-like structures were predominant in solution. No coil-like structures were detected at temperatures close to 25 °C. Therefore, it could be deduced that the temperature close to 25 °C is favorable for POXs to self-assemble at the sensing layer.

Based on these data, it was decided to use for non-biofouling layers only POX with a molecular weight of 2kDa (Figure 4b) at a temperature of 25 °C and dried in air.

### 3.4. Scanning Electron Microscopy (SEM)

**Figure 5:**
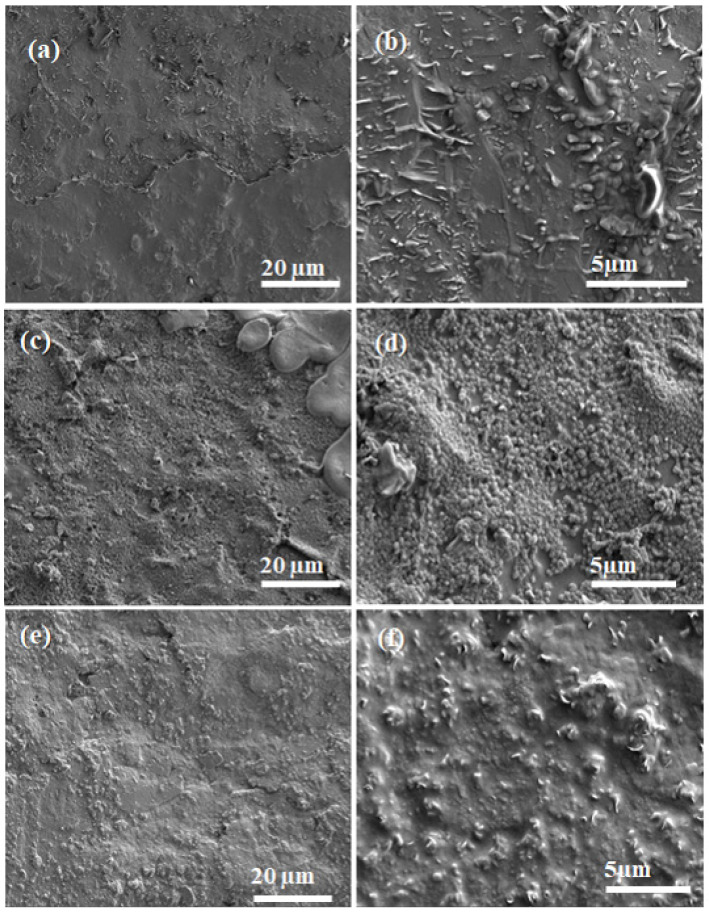
SEM image of G.C (**a**,**b**), PANI-deposited @G.C (**c**,**d**), and PANI@G.C with POX (**e**,**f**).

SEM was used to investigate the surface changes of G.C after the deposition of PANI and POX (Figure 5). SEM images of G.C electrodes (Figure 5a,b), a PANI-layer-deposited @G.C electrode (Figure 5c,d), and POX/PANI@G.C (Figure 5e,f) are presented. It is shown that the PANI layer completely covers the G.C surface. Moreover, the deposition of the POX layer is uniform, which is crucial in non-biofouling applications.

It must be emphasized that the deposition of chelating agents on top of the PANI did not change its morphology. This fact was explained by the similar chemical natures of these molecules.

## 4. Potentiometric Testing of Fe^2+^ and Fe^3+^ Ions

**Figure 6:**
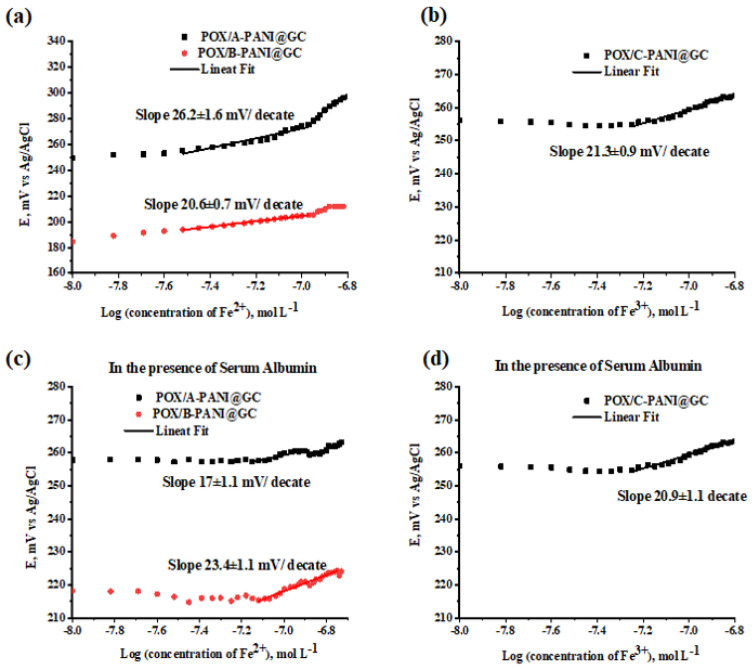
(**a**) Potentiometric detection of Fe^2+^ ions by POX/A-PANI@G.C and POX/B-PANI@G.C without BSA; (**b**) potentiometric detection of Fe^3+^ ion by POX/C-PANI@G.C without BSA; (**c**) potentiometric detection of Fe^2+^ ions by POX/A-PANI@G.C and POX/B-PANI@G.C with BSA; and (**d**) potentiometric detection of Fe^3+^ ion by POX/C-PANI@G.C with BSA.

Three types of chelating molecules were used for the chelation of either Fe^2+^ ions or Fe^3+^ ions, that is, 2,2-bipyridyl, 1,10-phenanthrolin-5-amine, and 8-hydroxyquinoline; see Table 1. The deposition of chelating agents was performed by drop-casting, and the fixation of the deposited chelating layer to PANI film was performed by cyclic voltammetry with a 50 mV/s scan rate in acetonitrile. The chelating layers, on one hand, were able to selectively detect iron ions, and on the other hand, the lower PANI layer was used only as an ion-to-electron transducer. The deposited non-biofouling POX layer protected the chelating layer from non-specific interactions. The results of the potentiometric measurements are presented in Figure 6. POX/A-PANI@G.C and POX/B-PANI@G.C were tested as sensing layers for the Fe^2+^ ions (Figure 6a), and POX/C-PANI@G.C was investigated as a sensing layer for Fe^3+^ ions (Figure 6b).

Before testing the developed sensing layers, it was decided to test PANI@G.C electrodes towards the presence of iron ions, and the results are presented in SI Figure S6. It was confirmed that PANI itself does not respond to the presence of iron ions. Moreover, to check the POX/A-PANI@G.C, POX/B-PANI@G.C, and POX/C-PANI@G.C sensing electrodes towards the presence of protons, the potentiometric response vs. pH was monitored, and the results are presented in Figure S7. It was confirmed that sensing layers developed by us were not sensitive towards protons and could be tested for the detection of iron ions.

After conditioning the sensing electrodes in a 0.1 M solution of NaCl (the solution was also purged with N_2_ gas to avoid the oxidation of Fe^2+^ to Fe^3+^), the potentiometric response of the POX/A-PANI@G.C vs. Fe^2+^ ions gave an average slope of 26.2 ± 1.6 mV/decade (*n* = 4) with a working range of Fe^2+^ ion concentrations from 5 nM to 5 µM. The theoretical Nernstian slope was 29.5 mV/decade. We could speculate that the lower value for the slope could be explained by the presence of a non-biofouling layer. The potentiometric response of the POX/B-PANI@G.C is presented in Figure 6a and has a slope value of 20.6 ± 0.7 mV/decade (*n* = 4) in the Fe^2+^ concentration range from 5 nM to 5 µM. From these measurements, it was concluded that POX/A-PANI@G.C is more sensitive toward Fe^2+^ ions compared to the POX/B-PANI@G.C. We have tested the sensitivity of the POX/A-PANI@G.C and POX/B-PANI@G.C towards Fe^3+^ ions, and the results are presented in Figure S8. It is obvious that these two sensing composite layers are not able to detect Fe^3+^ ions.

For Fe^3+^ detection, the POX/C-PANI@G.C was used, as presented in Figure 6b. The potentiometric response gave an average slope of 21.3 ± 0.9 mV/decade (*n* = 4) with a theoretical value of 19.6 mV/decade. The concentration range for Fe^3+^ ion detection was from 5 nM to 50 µM. A control experiment was performed with Fe^2+^ ions for POX/C-PANI@G.C, and the result is presented in SI, Figure S9. Indeed, the sensing layer of the POX/C-PANI@G.C electrode did not detect Fe^2+^ ions. Based on these results, we concluded that POX/C-PANI@G.C could be used as a promising selective detection layer for Fe^3+^ ions.

It is well-known that non-specific protein interactions with the sensing layer could lead to poor performance. In order to avoid it, the non-biofouling layer was attached to the surface. The performances of the POX/A-PANI@G.C, POX/B-PANI@G.C, and POX/C-PANI@G.C in the presence of BSA are presented in Figure 6c,d (and the EIS of POX/PANI@G.C with BSA is presented in Figure S2). When the POX/A-PANI@G.C and POX/B-PANI@G.C were tested for the sensing of Fe^2+^ ions in the presence of BSA, the average slopes of 17 ± 1.1 mV/decade (*n* = 4) and 23.4 ± 1.1 mV/decade (*n* = 4) were measured. The concentration range was shorter, from 50 nM to 50 µM, compared to the testing without BSA. On the other hand, the sensing performance of the POX/C-PANI@G.C electrode did not change in the presence of BSA, with an average slope of 20.9 ± 1.1 mV/decade (*n* = 4) (see Figure 6d). This is consistent with the known iron-complexing properties of BSA, especially to divalent ions, while the interaction of BSA with Fe^3+^ ions is significantly weaker in a lactoferrin-like manner (and requires hydrogen carbonate) and is less specific [40,41,42]. These results suggest that all three developed sensing electrodes work, even in the presence of BSA.

### Selectivity Testing in the Presence of Cu^2+^

**Figure 7:**
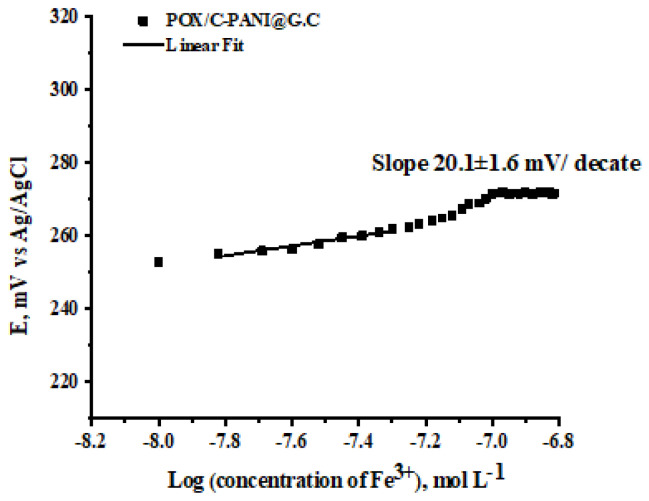
Potentiometric detection of Fe^3+^ ions by POX/C-PANI@G.C in the presence of Cu^2+^ ions.

To investigate the performance of the developed composite sensing layers, the potentiometric detection of Fe^3+^ ions was conducted in the presence of interfering ions, such as Cu^2+^. The results of the measurements are presented in Figure 7. This investigation was conducted for Fe^3+^ ions because Fe^2+^ ions could be oxidized to Fe^3+^ ions.

An ideal iron-sensing layer should be selective for Fe^3+^ ions in order to minimize the chelation of other biologically essential metal ions, such as Cu^2+^. The potentiometric response gave an average slope of 20.1 ± 1.6 mV/decade (*n* = 4) with a theoretical value of 19.6 mV/decade. The concentration range for Fe^3+^ ion detection was from 1 nM to 1 µM. When the concentration of the Fe^3+^ ions increased, the composite sensing layer stopped responding. Such behavior could be explained by the limitation connected with the transport of ions through the POX film.

## 5. Conclusions

Three types of chelating molecules were used in order to develop the sensing layer for the detection of Fe^2+^ or Fe^3+^ ions. It was shown that the POX/A-PANI@G.C is more sensitive toward Fe^2+^ ions compared to the POX/B-PANI@G.C. To detect Fe^3+^ ions, the POX/C-PANI@G.C could be used as a promising sensing layer. These sensing layers work in the presence of BSA and interfering ions. The whole concept of the layered sensing composite film is based on the idea that the PANI layer is an excellent candidate as an ion-to-electron transducer. On top of the PANI layer, the chelating layer is deposited, which is fixed by the electrochemical method. Finally, the non-biofouling layer is deposited by the drop-casting technique and is also fixed by the electrochemical method.

## Figures and Tables

**Table 1:** The names of the prepared sensing electrodes.

Deposited Chelating Molecules and POX		Names
2,2-Bipyridyl	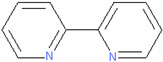	POX/A-PANI@G.C
1,10-Phenanthroline-5-amine	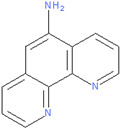	POX/B-PANI@G.C
8-Hydroxyquinoline	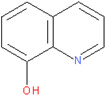	POX/C-PANI@G.C

## Data Availability

Not applicable.

## Supplementary Materials

The following supporting information can be downloaded at: https://www.mdpi.com/article/10.3390/bios12070446/s1, Figure S1: Electrochemical impedance spectroscopy of A-PANI@G.C, B-PANI@G.C, and C-PANI@G.C; Figure S2: EIS of electrodes in the presence of BSA; Figure S3: Raman spectra of A-PANI2G.C; Figure S4: Raman spectra of B-PANI@G.C; Figure S5: Raman spectra of C-PANI@G.C; Figure S6: Potentiometric measurements of PANI layer without chelating molecules; Figure S7: Potentiometric response of different sensing layers vs. pH; Figure S8: Potentiometric response of POX/A-PANI@G.C and POX/B-PANI@G.C vs. Fe3+; Figure S9: Potentiometric response of POX/C-PANI@G.C vs. Fe2+.
