# Supplementary material for: Potentiometric Performance of Ion-Selective Electrodes Based on Polyaniline and Chelating Agents: Detection of Fe2+ or Fe3+ Ions
Source: Biosensors (Basel). 2022 Jun 23;12(7):446. doi: 10.3390/bios12070446 (PMC9313018; doi:10.3390/bios12070446)
Supplement: Supplementary file 1 [file biosensors-12-00446-s001.zip › biosensors-1766447-supplementary.pdf]

Supporting Information

Potentiometric performance of ion-selective-electrodes based on polyaniline and chelating agents: Detection of Fe^{2+} or Fe^{3+} ions

Rimeh Ismail[1], Ivana Šeděnková[1], Zulfiya Černochová[1], Iryna Romanenko[1], Ognen Pop-Georgievski[1], Martin Hrubý[1], Elena Tomšík[1*]

[1]Institute of Macromolecular Chemistry AS CR, Heyrovského nám. 2, 162 06 Prague 6, Czech Republic
tomsik@imc.cas.cz

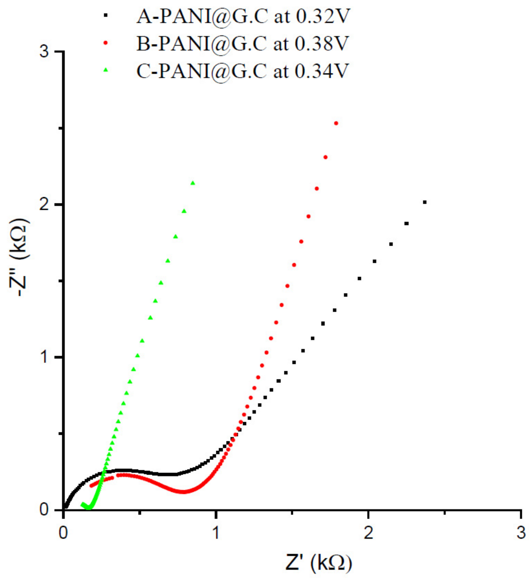

Figure S1. EIS in 0.1 M NaCl of A-PANI@G.C, B-PANI@G.C, and C-PANI@G.C.

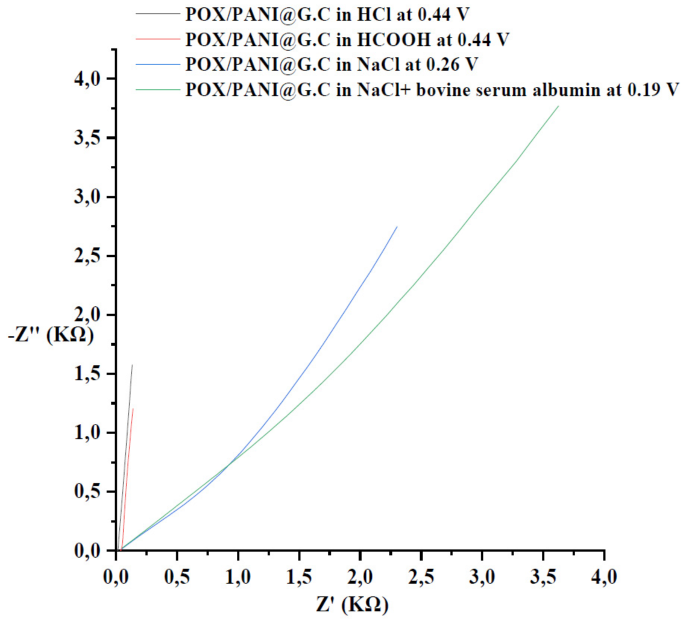

Figure S2. EIS POX/PANI@G.C in different electrolytes and with BSA.

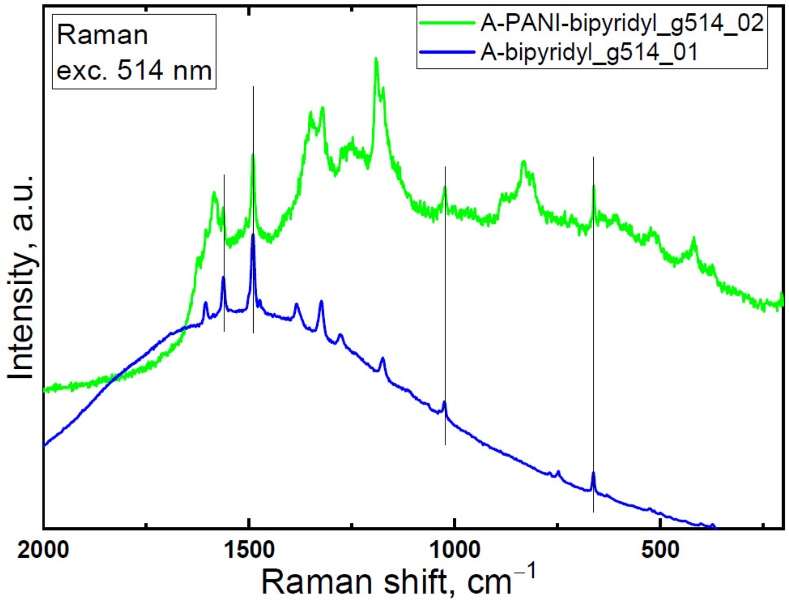

Figure S3. Raman spectra, excitation line 514 nm, of A-PANI @G.C.

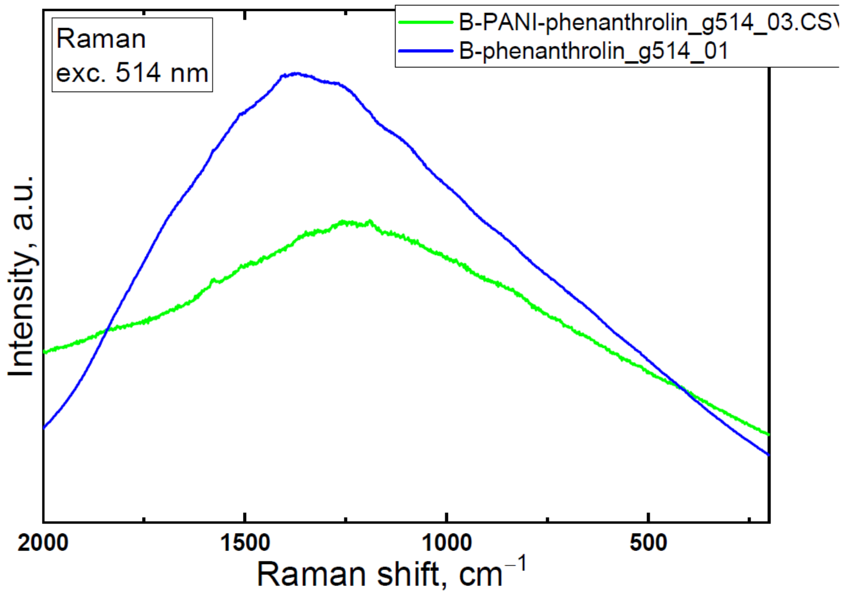

Figure S4. Raman spectra, excitation line 514 nm, of B -PANI @G.C.

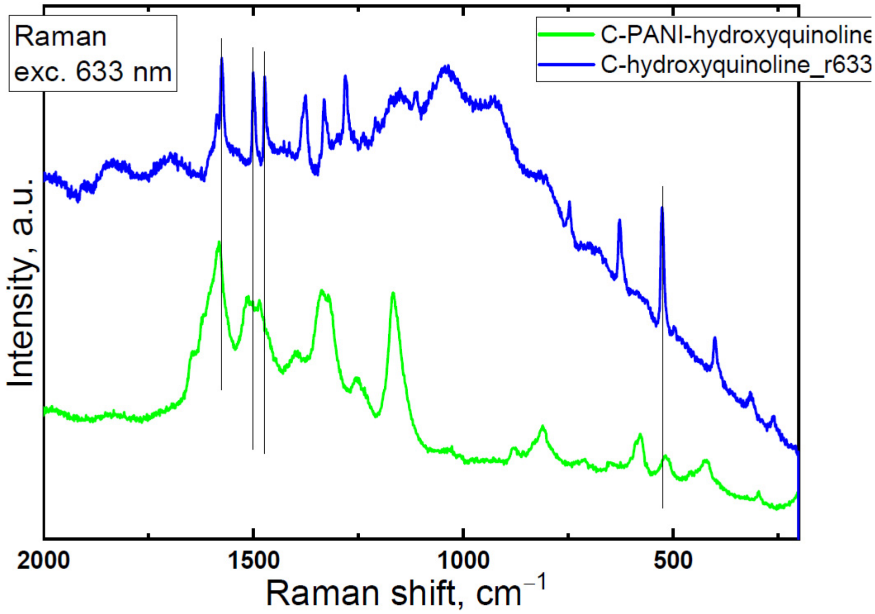

Figure S5. Raman spectra, excitation line 633 nm, of C-PANI@G.C.

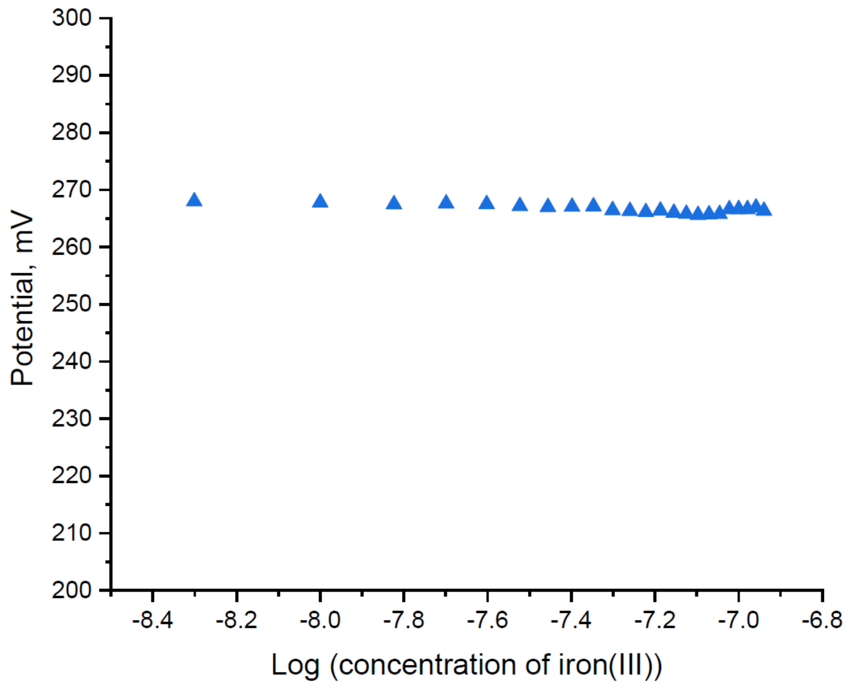

Figure S6. Potentiometric measurements of PANI@G.C layer without the presence of chelating molecules.

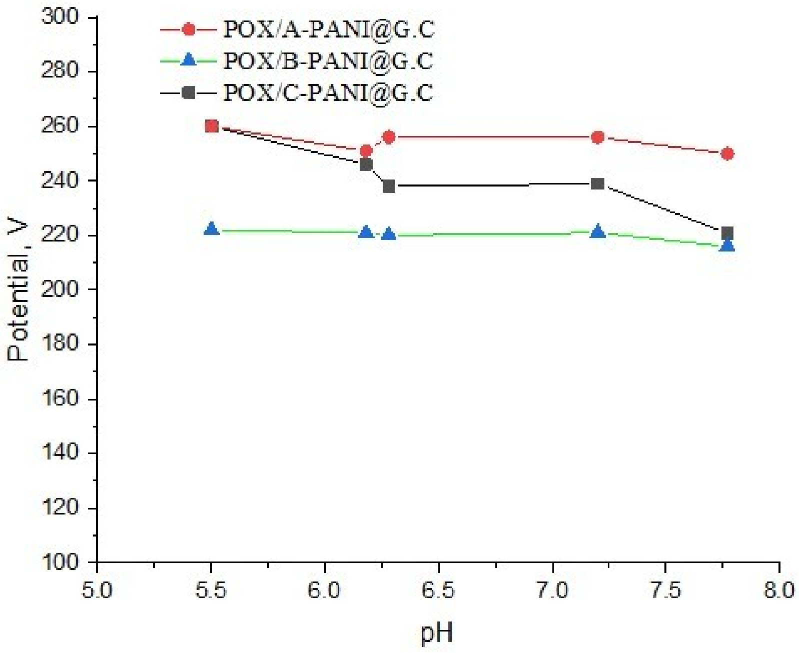

Figure S7. Potentiometric response of different sensing layers (different chelating agents) vs pH.

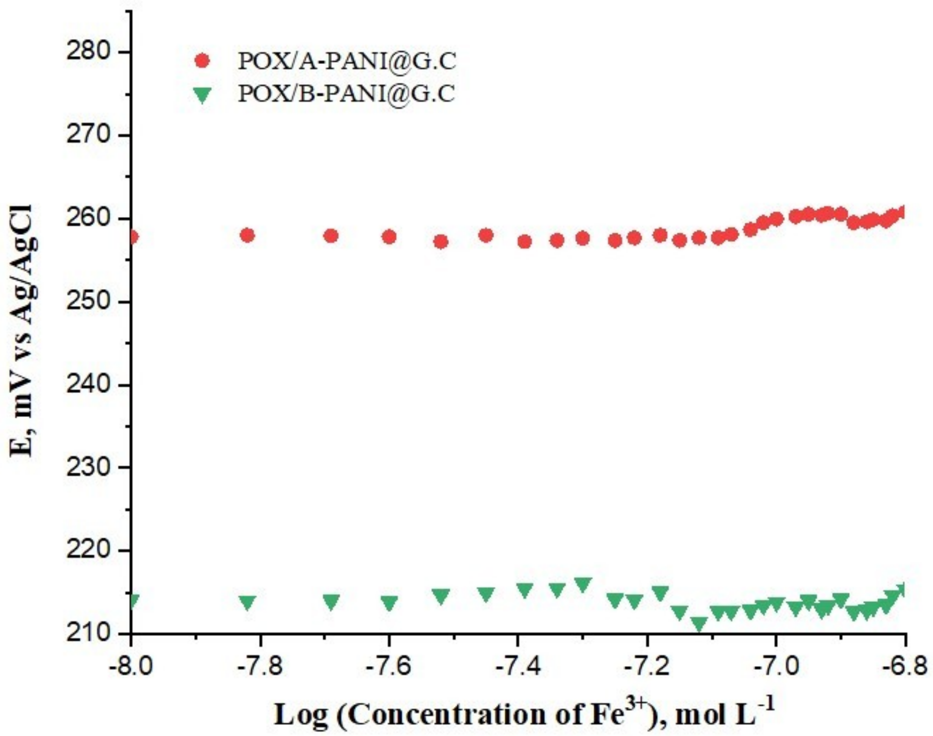

Figure S8. Potentiometric response (Fe^{3+} concentration vs E) of POX/A-PANI@G.C and POX/B-PANI@G.C sensing layers in 0.1 M NaCl.

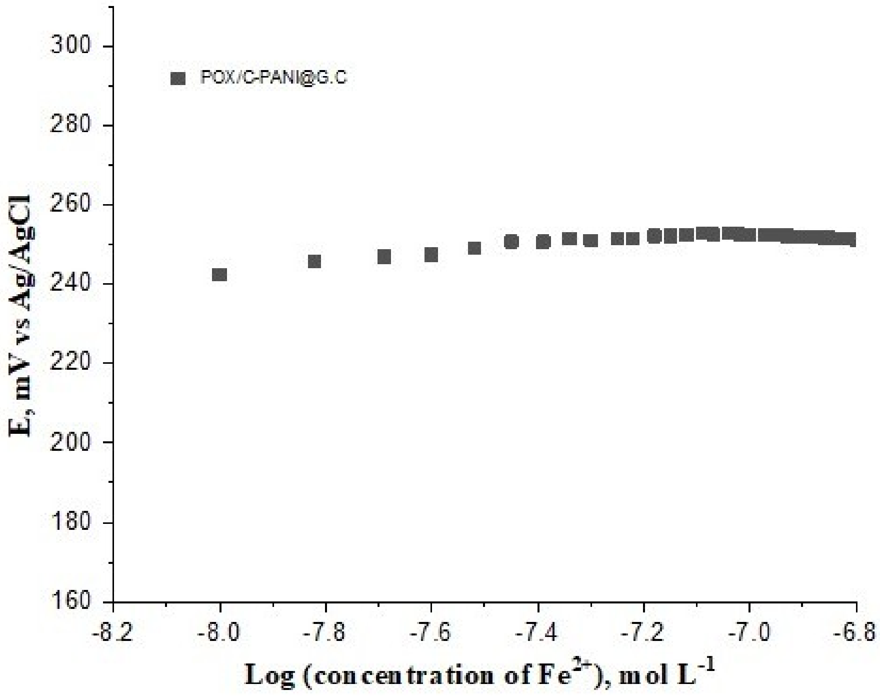

Figure S9 Potentiometric response (Fe^{2+} concentration vs E) of POX/C-PANI@G.C sensing layers in 0.1 M NaCl.
